# Human amnion-derived mesenchymal stem cells attenuate acute lung injury in two different acute lung injury mice models

**DOI:** 10.3389/fphar.2023.1149659

**Published:** 2023-06-14

**Authors:** Yuxuan Wu, Hao Sun, Lianju Qin, Xiaomin Zhang, Hao Zhou, Yao Wang, Lumin Wang, Meng Li, Jiayin Liu, Jinsong Zhang

**Affiliations:** ^1^ Department of Emergency, Jiangsu Province Hospital, The First Affiliated Hospital of Nanjing Medical University, Nanjing, Jiangsu, China; ^2^ State Key Laboratory of Reproductive Medicine, Center of Clinical Reproductive Medicine, The First Affiliated Hospital of Nanjing Medical University, Nanjing, China; ^3^ Department of Emergency, Jiangnan University Medical Center, Wuxi, China

**Keywords:** acute lung injury, acute respiratory distress syndrome, lipopolysaccharide, paraquat, human amnion-derived mesenchymal stem cells, NF-κB signaling pathway

## Abstract

Acute lung injury (ALI) is one of the most common clinical emergencies with limited effective pharmaceutical treatment in the clinic, especially when it progresses to acute respiratory distress syndrome (ARDS). Currently, mesenchymal stem cells (MSCs) exhibit specific superiority for ALI/ARDS treatment. However, stem cells from different sources may result in controversial effects on similar disease conditions. This study aimed to determine the effects of human amnion-derived mesenchymal stem cells (hAMSCs) on two different ALI mice model. The administered hAMSCs effectively accumulated in the lung tissues in all hAMSC-treated groups. Compared with the model and 1% human serum albumin (HSA) groups, high-dose hAMSCs (1.0 × 10^6^ cells) group significantly alleviated alveolar-capillary permeability, oxidative stress, inflammatory factors level and histopathological damage. In addition, the NF-κB signaling pathway is one of the key pathways activated during lipopolysaccharide (LPS) or paraquat (PQ)-induced lung injury. Our results indicated that hAMSCs (1.0 × 10^6^ cells) obviously inhibited the expression of p-IKKα/β, p-IκBα, and p-p65 in the lung tissue (*p* < 0.05). The high-dose (HD) hAMSC treatment exerted beneficial therapeutic effects on ALI mice models without detectable adverse reactions. The therapeutic effect of hAMSCs might involve NF-κB signaling pathway inhibition. hAMSC treatment is a potential candidate therapy for ALI.

## 1 Introduction

Acute lung injury (ALI) is a group of syndromes characterized by increased alveolar-capillary permeability and hypoxemia. Acute respiratory distress syndrome (ARDS) is the most severe form of ALI and is usually caused by a variety of complex factors, including pneumonia, sepsis, trauma, and chemical poisoning. ARDS is characterized by an inflammatory response, pulmonary epithelial cell apoptosis, oxidative stress, impaired clearance of alveolar fluid, and imbalance in the coagulation-fibrinolytic system (Ranieri et al., 2012; Meyer et al., 2021). The interaction between all pathological processes in ARDS induces an uncontrollable inflammatory response that ultimately leads to pulmonary fibrosis and even death. The release of massive inflammatory factors activates the NF-κB signaling pathway in ALI. Recently, with the development of medical technology, a series of supporting treatments, including lung-protective ventilation, fluid-conservative therapy and extracorporeal membrane oxygenation (ECMO), have improved the prognosis of ALI and ARDS (Fan et al., 2018; Meyer et al., 2021). However, the high lethality of ARDS (Bellani et al., 2016; Abe et al., 2018; Papazian et al., 2019) still urges researchers to explore more effective solutions.

In recent years, stem cell therapy has become a hot topic worldwide, mainly applied in musculoskeletal diseases, trauma, cornea, cardiovascular diseases and rheumatic system diseases. Mesenchymal stem cell (MSC)-based treatment exerts satisfactory therapeutic effects on ARDS (Johnson et al., 2017; Boyle et al., 2019; Yang et al., 2019). In preclinical studies, MSCs can be recruited directionally to damaged tissues and exhibit potential therapeutic effects on both infectious and noninfectious lung injury (Hass et al., 2011; Ryan et al., 2019). Preclinical studies have suggested that *in vivo* administration of MSCs exerts anti-inflammatory and anti-apoptotic effects, enhances epithelial and endothelial cell recovery, promotes microbial and alveolar fluid clearance, and reduces lung and distal organ injuries in the treatment of ARDS (Xuan et al., 2017; Qin and Zhao, 2020; Tu et al., 2022). On this basis, few clinical studies have demonstrated the safety assessment and improvement of inflammatory markers in the treatment of ARDS patients (Wilson et al., 2015; Yip et al., 2020; Monsel et al., 2022). Due to the lack of control group and small sample size, the results cannot be further analyzed.

Among them, human amnion-derived mesenchymal stem cells (hAMSCs) are the main biological raw material of the placental amniotic membrane. The advantages of hAMSCs increase the possibility of clinical treatment, such as no ethical problems, sufficient supply, and multidirectional differentiation ability (Liu et al., 2021a). However, the current research on hAMSCs is limited. Published studies have shown that hAMSCs can obtain some features of epithelial cells *in vitro* and *in vivo*, such as the expression of cystic fibrosis transmembrane conduction adjustment factor (CFTR) (Carbone et al., 2014) and surface-active protein, which could reduce pulmonary fibrosis and inflammation and promote lung function recovery. By modulating the B-cell response, hAMSCs may contribute to blunting of the chronification of lung inflammatory processes with a consequent reduction in the progression of fibrotic lesions (Cargnoni et al., 2020). And hAMSCs could alleviate paraquat-induced pulmonary fibrosis by inhibiting the inflammatory response and improve survival rate in rats (He et al., 2020; Gong et al., 2022). These conclusions have been confirmed in the model of bleomycin-induced and paraquat-induced pulmonary fibrosis. However, before hAMSCs can be used for the clinical therapy of acute and chronic lung diseases, a large amount of biological and clinical data must be collected. As a potential candidate for ALI treatment, hAMSCs must be explored in in-depth preclinical studies.

The purpose of this study was to evaluate the safety and efficacy of hAMSCs in the treatment of acute lung injury in mice caused by two different pathogenic factors.

## 2 Materials and methods

### 2.1 Animals

Male C57BL/6J mice (8–9 wk old) were purchased from Charles River Laboratories (Beijing, China). Animal experiments were carried out in accordance with the guidelines established by the Institutional Animal Care and Use Committee of Nanjing Medical University (NMU; Jiangsu, China) (Permit Number: IACUC-1901054).

### 2.2 hAMSCs

hAMSCs were prepared at the State Key Laboratory of Reproductive Medicine, The First Affiliated Hospital of Nanjing Medical University, Jiangsu Province Hospital (Liu et al., 2021b), a Good Manufacturing Practice (GMP)-compliant laboratory according to the national principles. Also, the collection of human amniotic membrane and their use were conducted under the guidelines and with the approval of the first affiliated hospital with Nanjing medical university (2012-SR-128). All patients provided written informed consent to the respective use of their tissues. hAMSCs were isolated and characterized according to previous reports(Liu et al., 2021a; Qin et al., 2022). Detailed steps can be found in Supplementary Materials.

### 2.3 ALI induction and treatment

Using non-invasive airway inhalation anesthesia, male C57BL/6J mice were administered a single dose of 0.02 mg paraquat (PQ) or 0.1 mg lipopolysaccharide (LPS) (Sigma‒Aldrich, MO, United States; PQ or LPS diluted in 50 μL of sterile saline buffer) per mouse via intratracheal aerosolization (Model IAIC micro sprayer, High Pressure Syringe Model FMJ-250, Penn-Century, PA, United States) (Zhang et al., 2019a; Sun et al., 2020). Control animals received 50 μL of saline after isoflurane inhalation anesthesia. hAMSCs were administered via the tail vein at 4 h after chemical exposure, and control animals were injected with an equal volume of 1% human serum albumin (HSA). As described above, the mice were harvested 3 days after PQ or LPS exposure. Animal was randomly assigned to different groups (8 mice/group): the normal saline group (NS), the ALI group (LPS or PQ), the 1% human serum albumin group (LPS-HSA or PQ-HSA), the medium-dose group (LPS-MD or PQ-MD, 0.5 × 10^6^ cells), and the high-dose group (LPS-HD or PQ-HD, 1.0 × 10^6^ cells). According to the animal model lung injury measurements of American Thoracic Society (Matute-Bello et al., 2011), lung injury was assessed by measuring the inflammatory cell count and polymorphonuclear (PMN) percentage in bronchoalveolar lavage fluid (BALF) and the lung, alveolar-capillary permeability and lung injury scores after hematoxylin and eosin (HE) staining.

### 2.4 Measurement of total protein levels in BALF

To evaluate alveolar-capillary permeability in the lung, BALF supernatants were collected, and the total protein level (μg/mL) of BALF was measured by the bicinchoninic acid (BCA) method (Thermo Fisher Scientific, MA, United States).

### 2.5 Oxidative stress test

To determine the activity of superoxide dismutase (SOD) in BALF, the Superoxide Dismutase Detection Kit was used. A total glutathione/oxidized glutathione assay kit was used to measure GSSG in serum. These kits were purchased from Nanjing Jiancheng Bioengineering Institute (Nanjing, China). The assay was conducted according to the manufacturer’s instructions.

### 2.6 Sample preparation and flow cytometry (FCM)

According to our previous study (Zhang et al., 2019b), we isolated and extracted cells from BALF samples and the right lung lower lobe. The percentage of neutrophils present in the airways or lung was analyzed by FCM. The collected cells were stained with the following monoclonal anti-mouse antibodies: Gr1-PE (RB6–8C5) and Ly6G-APC (1A8) (Biolegend, CA, United States). The cells were stained for 15 min on ice before being washed and analyzed on a FACSVerse flow cytometer (BD, NJ, United States) using FlowJo V10.0.7 software (Tree Star, Ashland, United States). Gr-1^high^ Ly6G^+^ cells were considered neutrophils.

### 2.7 Cytokine and protein analysis

The levels of the cytokines (IL-1β, IL-6 and TNF-α) in the BALF were measured by ELISA. The mRNA levels of cytokines in the lungs were measured by quantitative real-time polymerase chain reaction. Detailed steps can be found in Supplementary Materials.

### 2.8 Western blot analysis

The expression of these pathway-related proteins in the lungs was detected by Western blot analysis. Detailed steps can be found in Supplementary Materials.

### 2.9 Histological analysis

The left lung lobe was fixed with 4% paraformaldehyde, embedded in paraffin, and prepared into 4-μm-thick sections. The sections were stained with HE and then scanned by a digital pathology section scanning system (Leica, ScanScope CS2, United States). The severity of lung injury was assessed according to the animal model lung injury scoring system of the American Thoracic Society (Matute-Bello et al., 2011).

### 2.10 Immunofluorescence analyses

The lung sections were also used for immunofluorescence (IF) staining (p-IκB, p-p65). The intensity of IF was detected by a fluorescence microscope (Leica, Thunder Imager, GER) and subsequently analyzed with ImageJ software (National Institutes of Health, Bethesda, Maryland, United States). The primary antibodies included p-IκB and p-p65 (#9936T, CST, Boston, United States). The secondary antibody was Alexa FluorTM 488 goat anti-rabbit IgG (H + L) (#A11008, Thermo Fisher Scientific, United States).

### 2.11 Statistics

All data were analyzed with GraphPad Prism 5 (GraphPad Software Inc., CA, United States). The data are presented as the mean ± S.D. for all experiments. Differences between groups were assessed by Student's t test or one-way analysis of variance (ANOVA) followed by Bonferroni’s multiple comparison test. All tests of statistical significance were two-sided, and statistical significance was set at *p* < 0.05.

## 3 Results

### 3.1 hAMSC characterization

**FIGURE 1:**
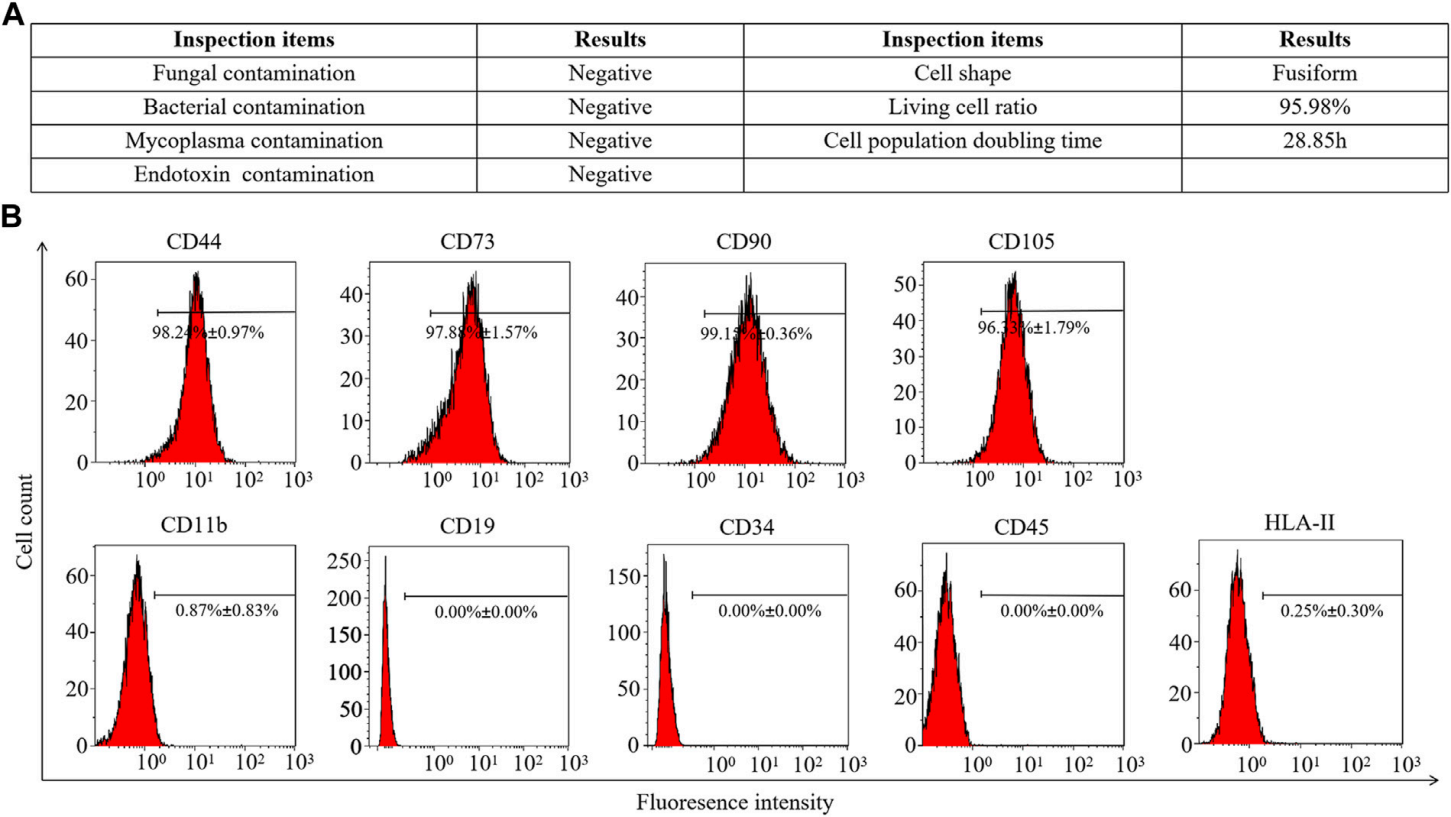
Identification of hAMSCs. **(A)** The general biosafety and viability of hAMSCs met the quality standard. **(B)** The expression of hAMSC surface antigens.

Fifth-passage hAMSCs (hAMSCs/P5) from the AMSC10 cell line were freshly prepared. The harvested hAMSCs/P5 exhibited high cell viability (≥95%). Meanwhile, bacterial contamination and *mycoplasma* infection were both eliminated (Figure 1A). To assess cell quality, the expression of mesenchymal and hematopoietic markers on hAMSCs/P5 was detected by FCM. As shown in Figure 1B, hAMSCs/P5 positively expressed mesenchymal markers (CD44 (98.24% ± 0.97%), CD73 (97.88% ± 1.57%), CD90 (99.15% ± 0.36%) and CD105 (96.33% ± 1.79%)) and negatively expressed hematopoietic markers (CD11b (0.87% ± 0.83%), CD19 (0.00% ± 0.00%), CD34 (0.00% ± 0.00%), CD45 (0.00% ± 0.00%) and HLA-DR (0.25% ± 0.30%)). Overall, the hAMSCs prepared in this study were of high quality.

### 3.2 Safety evaluation of hAMSCs

To assess the safety of hAMSCs application *in vivo*, different doses of cells were transplanted into mice by intravenous injection. As shown inSupplementary Figure S1A, the administration of 0.1 × 10^6^, 0.5 × 10^6^, and 1.0 × 10^6^ cells did not induce any death. When the single administration dose of hAMSCs exceeded 2.0 × 10^6^ cells/200 μL, some adverse reactions were observed in treated mice, such as shallow, rapid breathing irritability and even death. The mortality rates of mice that were administered 2.0 × 10^6^ cells or 4.0 × 10^6^ cells were 30.00% and 100.00%, respectively. Moreover, a high concentration of hAMSCs might increase the risk of pulmonary embolism. Therefore, three doses of hAMSCs (0.1 × 10^6^ cells, 0.5 × 10^6^ cells and 1.0 × 10^6^ cells) were selected to further evaluate the safety of hAMSCs in model mice with ALI. Compared with the saline group, mice treated with the three doses of hAMSCs (0.1 × 10^6^ cells, 0.5 × 106 cells, 1.0 × 10^6^ cells) showed no health problems and generally survived (Supplementary Figures S1B, C). Moreover, no significant body weight changes were observed for 3 days (Supplementary Figures S1D–F).

### 3.3 Distribution of hAMSCs in ALI mice

**FIGURE 2:**
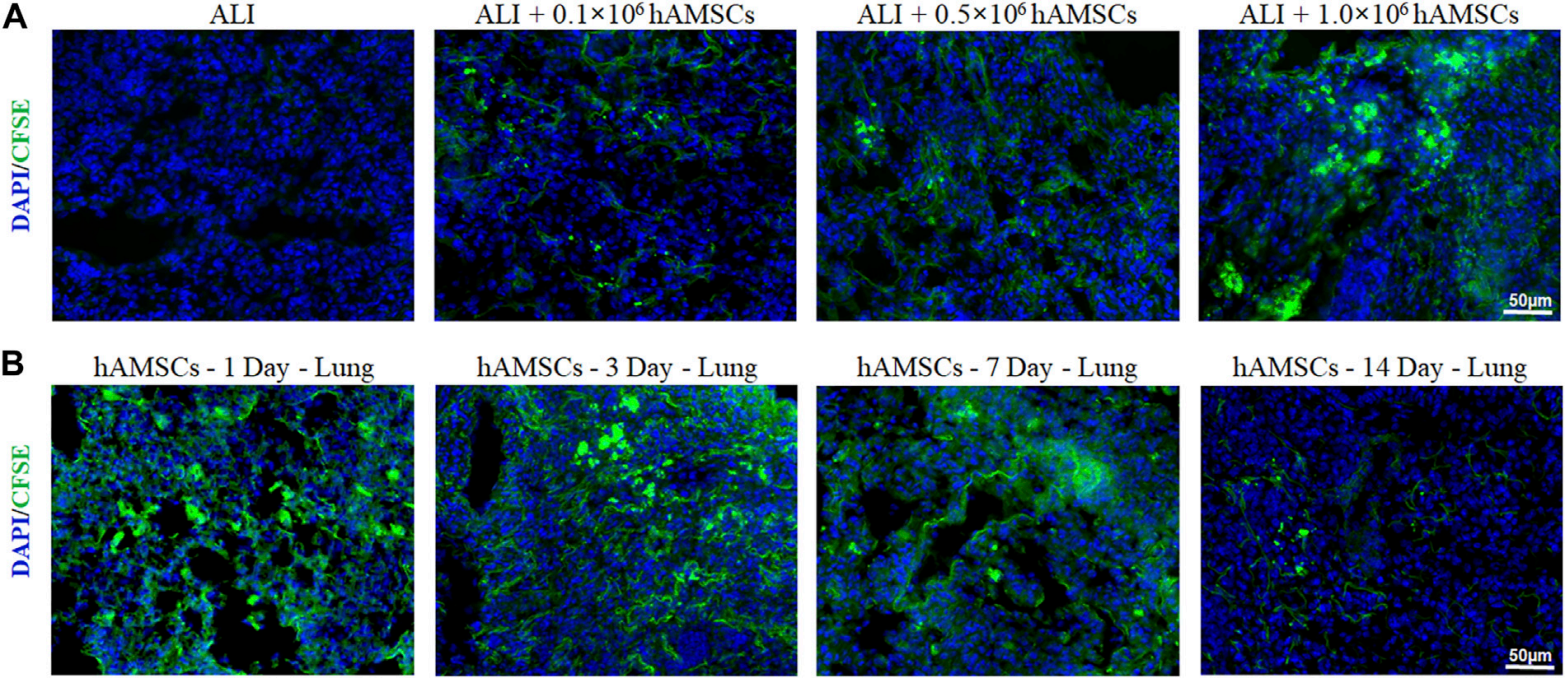
Distribution of hAMSCs. **(A)** Distribution of CFSE-labeled hAMSCs in the lung in the different dose groups, as determined by fluorescence microscopy. **(B)** Distribution of CFSE-labeled hAMSCs in the lung at 1, 3, 7, or 14 days after hAMSC treatment, as observed by fluorescence microscopy.

To determine the lung-targeting distribution of hAMSCs *in vivo*, the hAMSCs labeled with carboxyfluorescein succinimidyl amino ester (CFSE) was observed by fluorescence microscopy. As shown in Figure 2A, the fluorescence signal could be observed in all ALI groups 3 days after injection with different doses of hAMSCs. The highest fluorescence intensity was detected in the group treated with the highest dose of hAMSCs (1.0 × 10^6^ cells) (Figure 2A). Moreover, the residence time of hAMSCs in the lung was explored. As shown in Figure 2B, the green signal of hAMSCs was detected in the lung for a long time (14 days), and the attenuation of the fluorescence signal was slow during the first week. These results suggested that hAMSCs mainly migrated to the lung tissue in ALI mice.

### 3.4 hAMSCs ameliorated ALI induced by LPS and PQ

We evaluated the therapeutic effect of hAMSCs on the LPS-ALI and PQ-ALI mouse models. Through intratracheal injection of LPS or PQ in C57BL/6 mice, two ALI mouse models were established, which exhibited neutrophilic alveolitis, disruption of the alveolocapillary membrane, and interstitial thickening. In this part, the therapeutic effect of hAMSCs was examined by measuring the inflammatory response, oxidative stress, and tissue damage.

#### 3.4.1 hAMSCs reduced lung edema and cell infiltration in ALI mice

**FIGURE 3:**
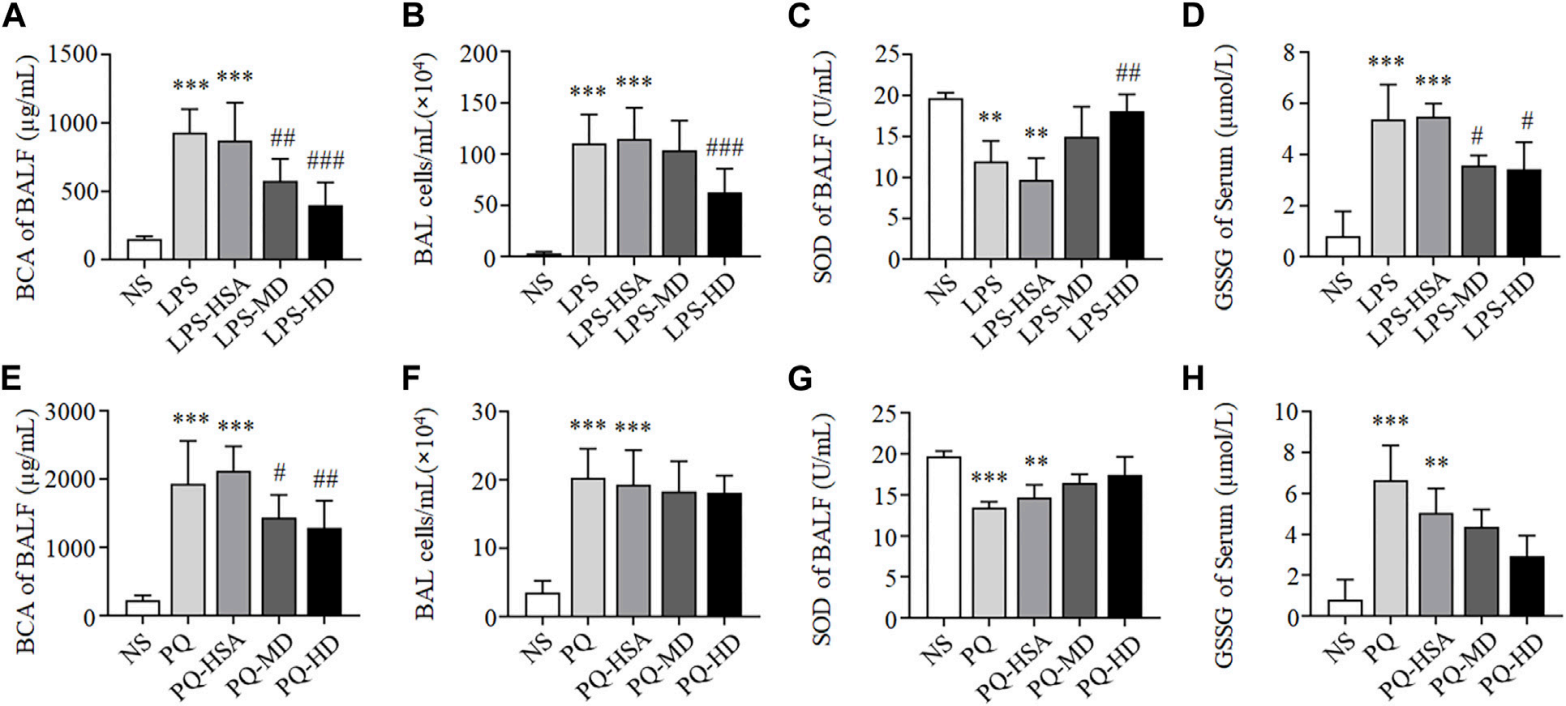
hAMSCs ameliorated the alveolar capillary barrier, cell infifiltration and oxidative stress in ALI mice. **(A, E)** Total protein concentration in BALF (A for LPS-ALI mice model, E for PQ-ALI mice model). **(B, F)** Quantifification of bronchoalveolar lavage (BAL) cells (B for LPS-ALI mice model, F for PQ-ALI mice model). **(C, G)** SOD activity in BALF (C for LPS-ALI mice model, G for PQ-ALI mice model). **(D, H)** GSSG in serum (D for LPS-ALI mice model, H for PQ-ALI mice model). *p < 0.05, **p < 0.01, and ***p < 0.001 compared to the NS group. #*p* < 0.05, ##*p* < 0.01, and ###*p* < 0.001 hAMSCs groups compared to LPS-HSA or PQ-HSA group (*n* = 8).

As shown in Figures 3A, B, compared to the NS group, the total BALF protein concentration and inflammatory cells in the LPS group and LPS-HSA group were significantly increased (*p* < 0.05). Moreover, the total BALF protein concentration and the inflammatory cells in the MD and HD groups were obviously lower than those in the LPS-HSA groups (Figures 3A, B).

Compared to the PQ-HSA group, the total BALF protein concentration significantly decreased in the PQ-MD and PQ-HD groups (*p* < 0.05) (Figure 3E). However, the number of inflammatory cells in BALF in the hAMSC-treated groups was slightly lower than that in BALF in the PQ-HSA groups (*p* > 0.05) (Figure 3F).

In summary, the therapeutic effects of hAMSCs on lung edema and cell infiltration in LPS-ALI mice were superior to those in PQ-ALI mice.

#### 3.4.2 hAMSCs inhibited oxidative stress in ALI mice

Oxidative stress in each groups was tested by SOD and GSSG detection. As shown in Figures 3C, D, compared to LPS and LPS-HSA groups, all groups with hAMSC transplantation showed higher SOD activity in BALF and lower GSSG levels in serum, especially LPS-HD group (*p* < 0.05).

Although we observed the lower SOD activity and the higher GSSG level in the PQ and PQ-HSA groups, the differences were not statistically significant in the hAMSC-treated groups (Figures 3G, H).

#### 3.4.3 hAMSCs inhibited the inflammatory response in ALI mice

**FIGURE 4:**
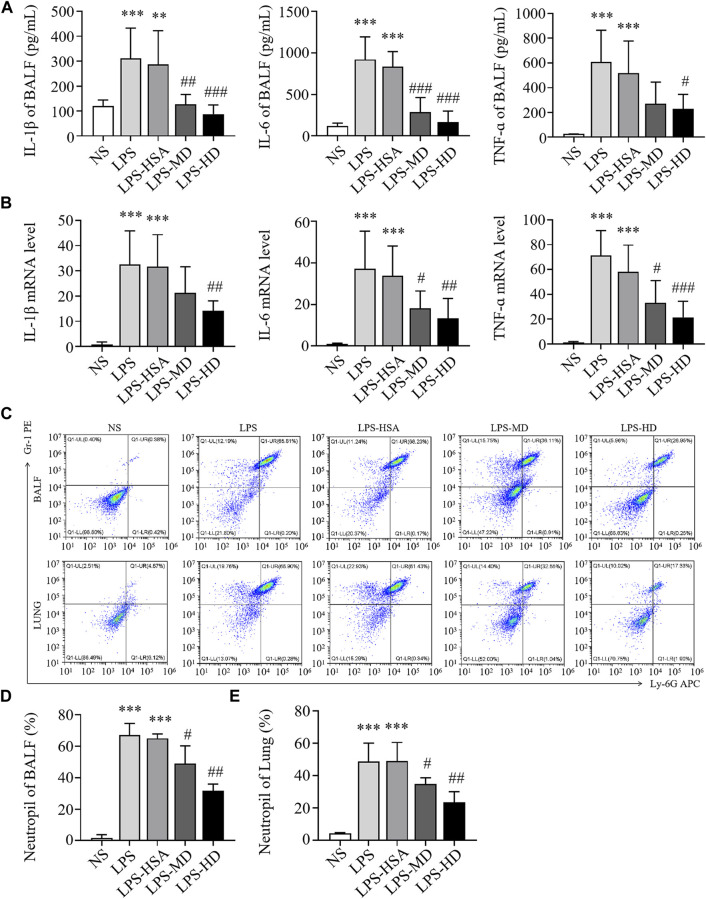
hAMSCs inhibited the inflammatory response in LPS induced ALI mice. **(A)** Levels of inflammatory cytokines (IL-1β, IL-6, and TNF-α) in BALF. **(B)** The mRNA levels of inflammatory cytokines (IL-1β, IL-6, and TNF-α) in the lung. **(C–E)** Neutrophil proportions in BALF and lung homogenates, as assessed by FCM. *p < 0.05, **p < 0.01, and ***p < 0.001 compared to the NS group. #*p* < 0.05, ##*p* < 0.01, and ###*p* < 0.001 hAMSCs groups compared to LPS-HSA group (*n* = 8).

To observe the anti-inflammatory effects of hAMSCs, we measured the levels of inflammatory factors in BALF and lung tissue. Compared to the LPS-HSA groups, the expression of proinflammatory factors (IL-1β, IL-6, and TNF-α) in BALF was downregulated in the hAMSC transplantation groups (Figure 4A). Similarly, the levels of IL-1β, IL-6 and TNF-α in lung tissue were significantly downregulated in the hAMSC transplantation groups, as assessed by RT‒PCR (Figure 4B). Furthermore, the percentage of PMNs in BALF and lung tissue in the LPS-ALI mouse model was evaluated. The results showed that LPS increased the PMN percentage in BALF and lung tissues. (*p* < 0.05). After hAMSC transplantation, the PMN percentage in BALF and lung tissue was significantly reduced (*p* < 0.05) (Figures 4C–E).

**FIGURE 5:**
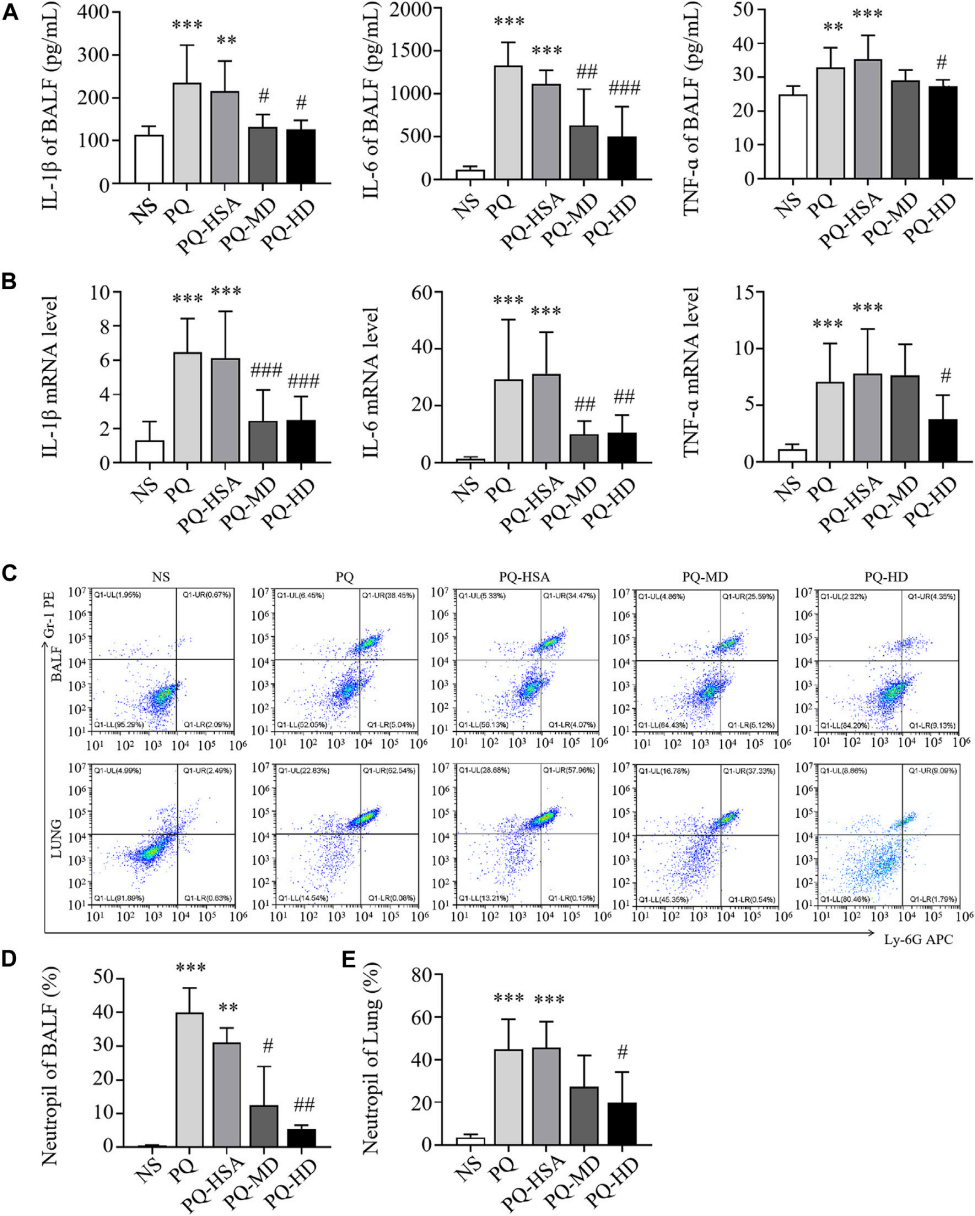
hAMSCs inhibited the inflammatory response in PQ induced ALI mice. **(A)** Levels of inflammatory cytokines (IL-1β, IL-6, and TNF-α) in BALF. **(B)** The mRNA levels of inflammatory cytokines (IL-1β, IL-6, and TNF-α) in the lung. **(C–E)** Neutrophil proportions in BALF and lung homogenates, as assessed by FCM. *p < 0.05, **p < 0.01, and ***p < 0.001 compared to the NS group. #*p* < 0.05, ##*p* < 0.01, and ###*p* < 0.001 hAMSCs groups compared to PQ-HSA group (*n* = 8).

In the PQ-ALI mouse model, inflammatory cytokine levels in BALF and lung tissue were also detected. Compared to the PQ-HSA group, the levels of IL-1β and IL-6 in BALF in the PQ-MD and PQ-HD groups were significantly decreased (Figure 5A). Similarly, the mRNA levels of IL-1β and IL-6 in lung tissue in the two hAMSC-treated groups were significantly lower than those in the HSA group (Figure 5B). TNF-α levels in BALF and lung tissue were significantly decreased in the HD groups and only showed a decreasing trend in the MD group (Figures 5A, B). Furthermore, the infiltration of PMNs in BALF and lung tissue in all groups was detected by FCM. PQ stimulated PMN bursts in BALF and lung tissue compared to NS (*p* < 0.05). The ratios of PMNs in BALF and lung tissue in the PQ-HSA group were 31.11% and 45.73%, respectively. Importantly, in the PQ-HD group, the PMN ratios in BALF and lung were reduced to 5.48% and 19.98%, respectively (Figures 5C–E).

#### 3.4.4 hAMSCs ameliorated LPS-induced or PQ-induced lung injury in mice

**FIGURE 6:**
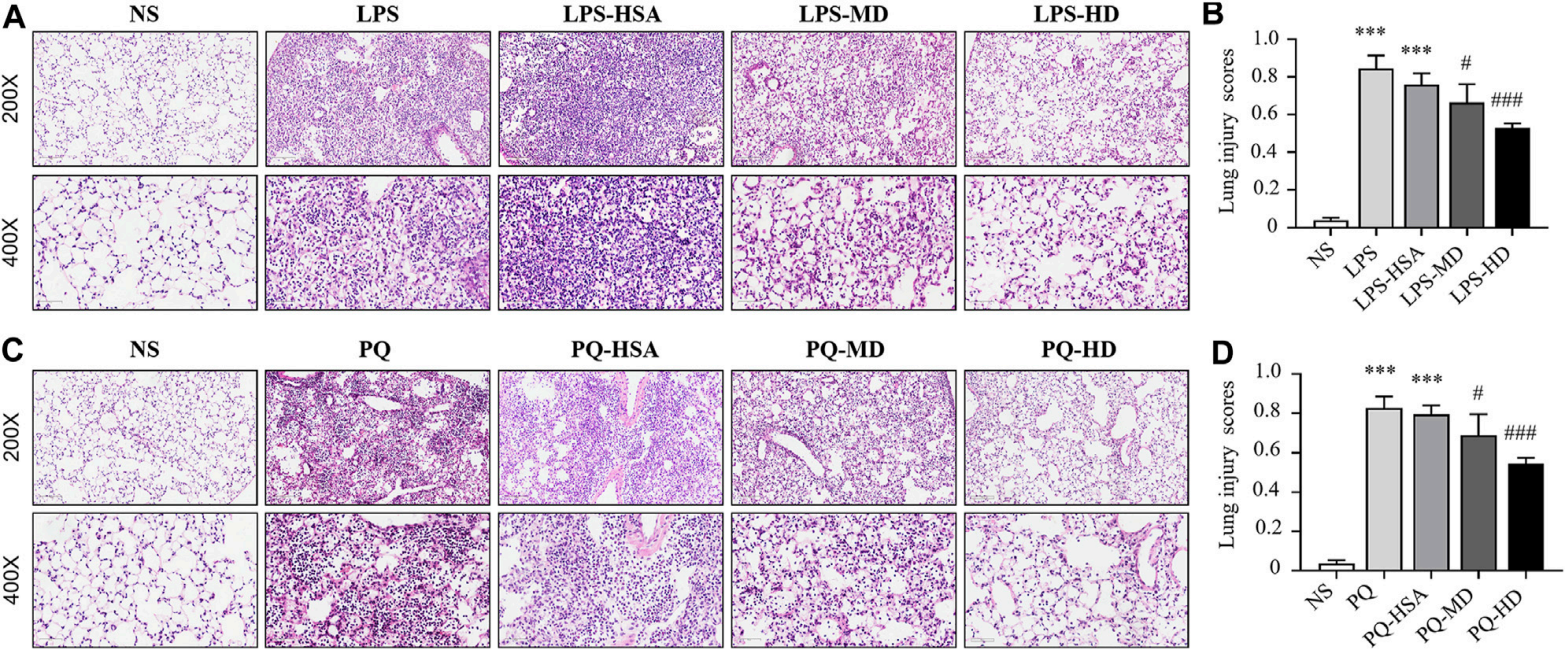
Mouse lung sections stained with HE and lung injury scores. **(A)** HE-stained slides of lung in LPS-induced ALI. **(B)** Histological lung injury scores measured with HE-stained slides in LPS-induced ALI. **(C)** HE-stained slides of lung in PQ-induced ALI. **(D)** Histological lung injury scores measured with HE-stained slides in PQ-induced ALI. Original magnification: top ×200, bottom ×400. The data are presented as the mean ± SD. *p < 0.05, **p < 0.01, and ***p < 0.001 compared to the NS group. #*p* < 0.05, ##*p* < 0.01, and ###*p* < 0.001 hAMSC groups compared with the PQ-HSA or LPS-HSA group.

Moreover, histological staining was implemented to examine the severity of lung injury and inflammatory infiltration. Compared to the NS group, the LPS and LPS-HSA groups showed significant anabatic structural damage and increased inflammatory cell infiltration. The hAMSC transplantation groups, especially the LPS-HD group, showed significant relief of lung tissue damage and inflammatory cell infiltration measured by the pathological lung injury scores (Figures 6A, B). These results indicated that hAMSCs could alleviate lung injury.

Compared to the PQ and PQ-HSA groups, hAMSC-treated groups showed alleviation of airspace inflammation and lung tissue damage (Figure 6C). The lung injury score was significantly decreased in PQ-HD group (Figure 6D). Consistently, a high dose (1.0 × 10^6^ cells) of hAMSCs showed an obviously enhanced therapeutic effect in ALI.

### 3.5 hAMSCs inhibited the NF-κB signaling pathway in the lungs of ALI mice

**FIGURE 7:**
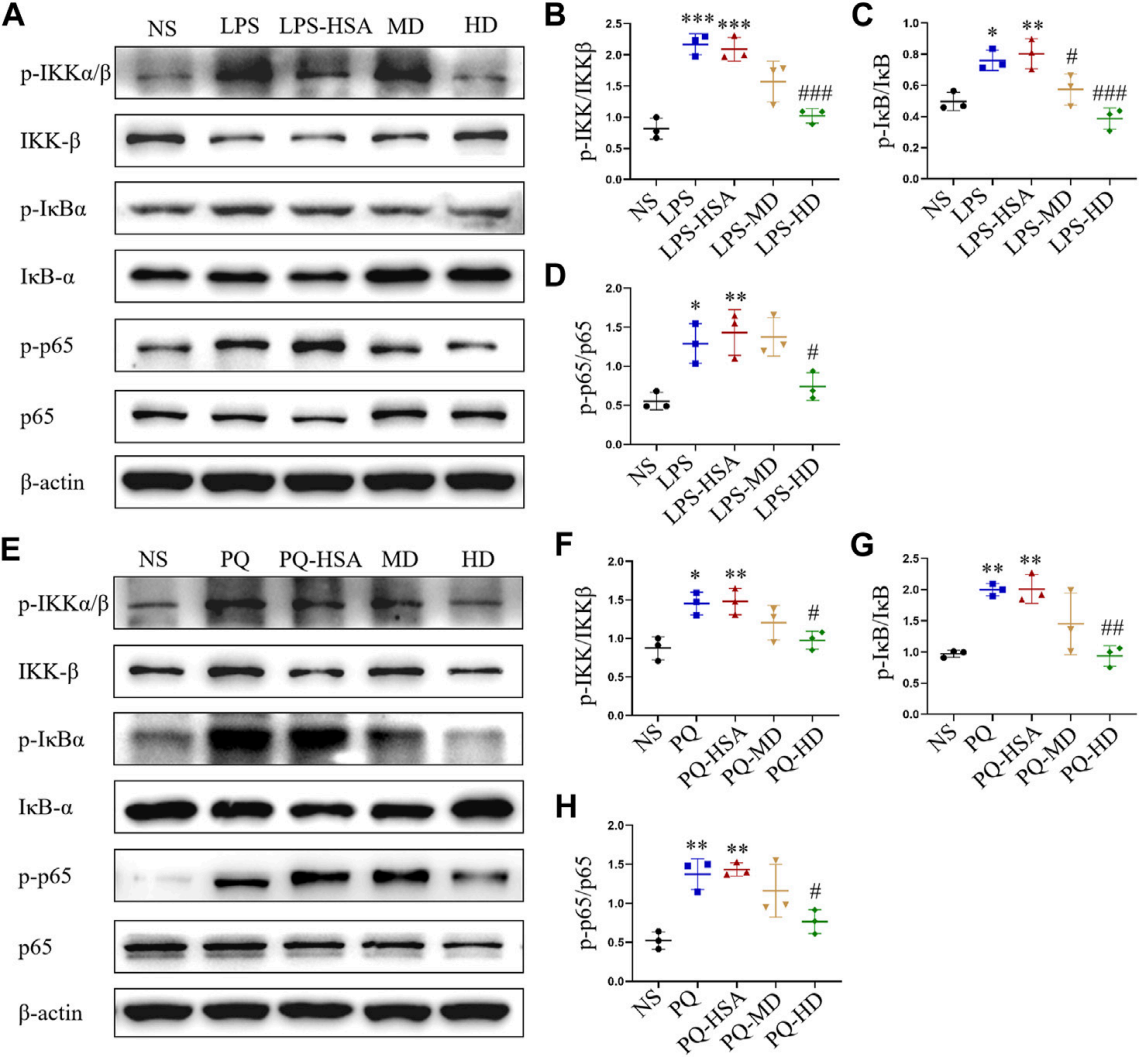
hAMSCs inhibited the NF-κB pathway in ALI mice induced by LPS or PQ. **(A)** The protein expression levels of p-IKKα/β, p-IκBα, p-p65, IKKβ, IκBα, p65, and β-actin in ALI mice induced by LPS. **(B–D)** The intensity of the protein bands in ALI mice induced by LPS was quantified. **(E)** The protein expression levels of p-IKKα/β, p-IκBα, p-p65, IKKβ, IκBα, p65, and β-actin in ALI mice induced by PQ. **(F–H)** The intensity of the protein bands in ALI mice induced by PQ was quantified. The data are presented as the mean ± SD. *p < 0.05, **p < 0.01, and ***p < 0.001 compared to the NS group. #*p* < 0.05, ##*p* < 0.01, and ###*p* < 0.001 hAMSC groups compared with the PQ-HSA or LPS-HSA group.

In the LPS-ALI and PQ-ALI mouse models, the expression levels of the phosphorylated forms of IKKα/β (p-IKKα/β), IκBα (p-IκBα) and NF-kB p65 (p-p65) were all upregulated (Figures 7A, E). Additionally, PQ and LPS induced an obvious increase in p-IKKα/β/IKKα/β, p-IκBα/IκBα, and p-p65/p65 at the protein level compared to the untreated group (Figures 7B–D, F–H). However, in the hAMSC-treated groups, the expression of p-IKKα/β, p-IκBα, and p-p65 was remarkably downregulated. Therefore, hAMSCs could inhibit the NF-κB signaling pathway by regulating the phosphorylation process of IKKα/β, IκBα and NF-κB p65.

**FIGURE 8:**
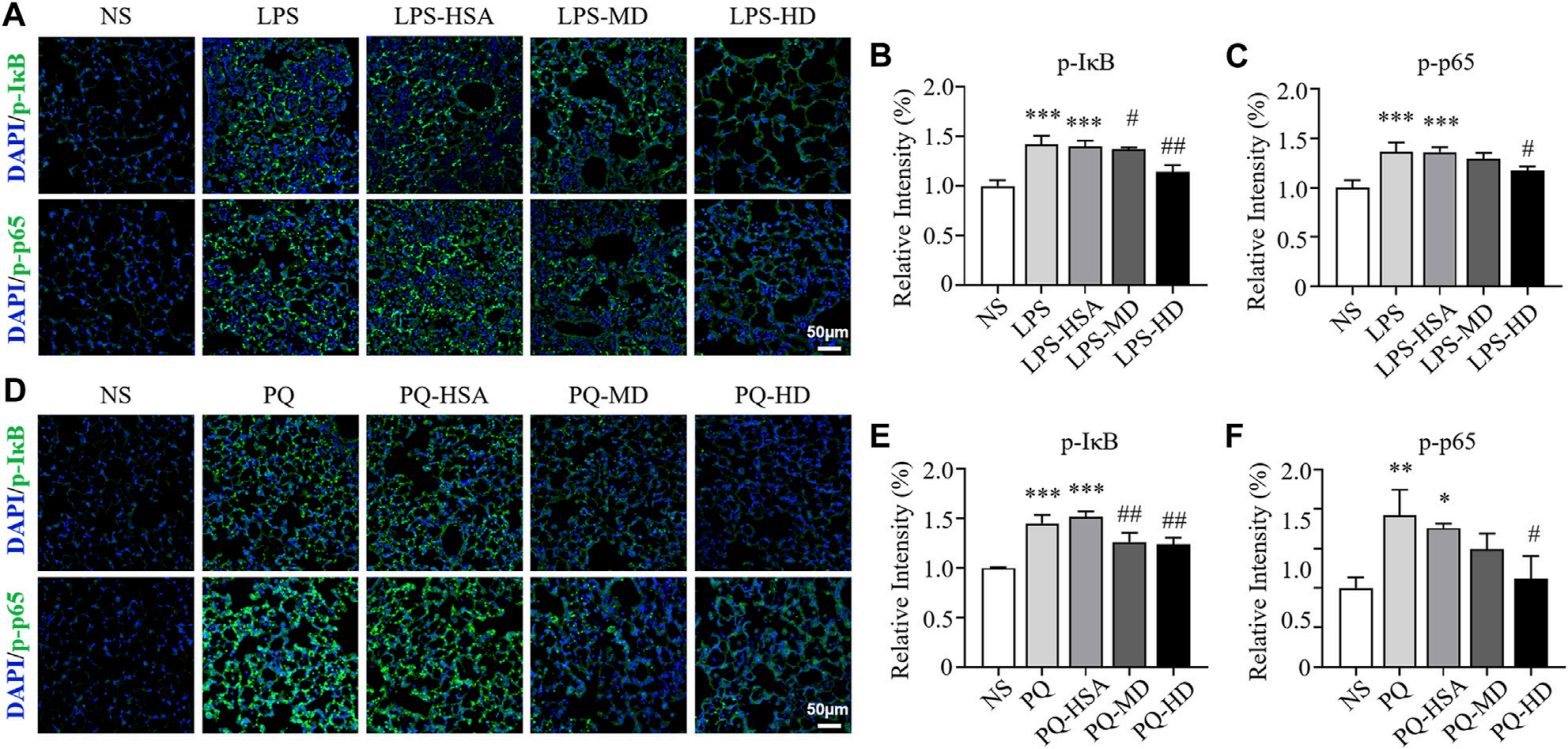
The effect of hAMSCs on the regulation of IκBα and NF-kB p65 phosphorylation in the LPS-ALI and PQ-ALI mouse models. **(A)** Immunofluorescence (p-IκBα, p-p65) analyses and **(B–C)** the relative optical densities of lung tissue sections of the LPS-ALI mouse model after the different treatments. **(D)** Immunofluorescence (p-IκBα, p-p65) analyses and **(E–F)** the relative optical densities of lung tissue sections of the PQ-ALI mouse model after the different treatments. Scale bars: 50 µm. The data are presented as the mean ± SD. *p < 0.05, **p < 0.01, and ***p < 0.001 compared to the NS group. #*p* < 0.05, ##*p* < 0.01, and ###*p* < 0.001 hAMSC groups compared with the PQ-HSA or LPS-HSA group.

Immunofluorescence (IF) staining was used to evaluate the expression of the phosphorylated forms of IκBα (p-IκBα) and NF-κB p65 (p-p65). In the LPS-ALI mouse model, the untreated group and HSA-treated group showed stronger fluorescence signals for both p-IκBα and p-p65 than the hAMSC-treated groups (Figures 8A–C). Then, the immunofluorescence images were semiquantitatively analyzed, and the fluorescence intensities of p-IκBα and p-p65 in the LPS-HD group were reduced to 81.42% and 86.45% of those in the LPS-HSA group, respectively (Figures 8A–C). For the PQ-ALI mouse model, the PQ-HD group showed the weakest fluorescence signals of p-IκBα and p-p65 in all groups (Figures 8D–F). The fluorescence intensities of p-IκBα and p-p65 in the PQ-HD group were 81.73% and 63.69% of those in the LPS-HSA group, respectively (Figures 8D–F). Based on our results, hAMSCs could inhibit the NF-κB signaling pathway.

## 4 Discussion

In this study, hAMSCs were used as a therapeutic treatment for ALI. The quality of hAMSCs was verified first. Our results showed that the general biosafety, viability, and growth characteristics of the prepared hAMSCs were all up to standard. Considering the vital importance of biosafety for therapeutic administration *in vivo*, the safety of hAMSCs has been varied. According to previous studies (Liu et al., 2021a; Qin et al., 2022), the tumorigenic potential and immune toxicity of 5.0 × 10^7^ hAMSCs/kg were negligible in C57BL/6 mice. Thus, different concentrations were examined to verify the *in vivo* safety of hAMSCs in this study. Consistent with previous reports (Liu et al., 2021b; Qin et al., 2022), 1.0×10^6^ did not exert detectable adverse reactions on mice in our study. When the single administration dose of hAMSCs exceeded 2.0 × 10^6^ cells/200 μL, some adverse reactions were observed in treated mice, such as shallow, rapid breathing irritability and even death. Moreover, a high concentration of hAMSCs might increase the risk of pulmonary embolism. Therefore, it is feasible to administer hAMSCs for ALI treatment at a dose of 1.0 × 10^6^ cells.

After intravenous injection, the targeted accumulation and retention of hAMSCs in the lung is a crucial factor in the therapeutic effect. Previous reports have shown that MSCs can distribute to inflamed lung tissue and enhance their potential efficacy (Cui et al., 2018; Liu et al., 2021a). According to our fluorescence results, hAMSCs were recruited to the lung and remained *in situ* for 14 days, especially in the PQ-induced ALI model group. This finding indicated that hAMSCs might have good therapeutic effects.

To evaluate the potential therapeutic effect of hAMSCs on ALI, we established a classical ALI model induced by LPS and an ALI model induced by PQ. In the two established ALI models, we observed an overwhelming inflammatory response, excessive oxidative stress, and detectable lung tissue injury, which are consistent with the main features of experimental ALI in mice (Matute-Bello et al., 2011; Zhang et al., 2019b). Compared with the model and 1% human serum albumin (HSA) groups, hAMSCs (1.0 × 10^6^ cells) significantly alleviated alveolar-capillary permeability, oxidative stress, and histopathological damage. In addition, hAMSCs also effectively decreased the levels of IL-Iβ, IL-6, and TNF-ɑ and polymorphonuclear (PMN) cell counts in the bronchoalveolar lavage fluid (BALF) and lung tissue of mice with ALI. Interestingly, the therapeutic effect of hAMSCs in LPS-ALI model was better than that in PQ-ALI model. There might be several reasons for the different treatment effect. Firstly, the mechanism of paraquat induced acute lung injury is more complex than LPS. LPS mainly acts as an endotoxin to activate macrophages and release proinflammatory factors. Subsequently, neutrophils are continuously activated and recruited to the lung by chemokine induction. Then, inflammation aggravates to form ALI/ARDS (Matute-Bello et al., 2011; Xiao et al., 2020). However, PQ is a thixotropic bipyridyl herbicide with high toxicity. In addition to inflammatory damage, paraquat also has direct contact toxicity and lipid peroxidation. These induces cellular damage to the alveolar epithelium and vascular endothelium, as well as neutrophil infiltration and an excessive inflammatory response (Zhang et al., 2019a; Sun et al., 2020). Secondly, the toxic effect of PQ may directly affect the activity of hAMSCs and reduce the therapeutic efficacy (Dinis-Oliveira et al., 2008).

**FIGURE 9:**
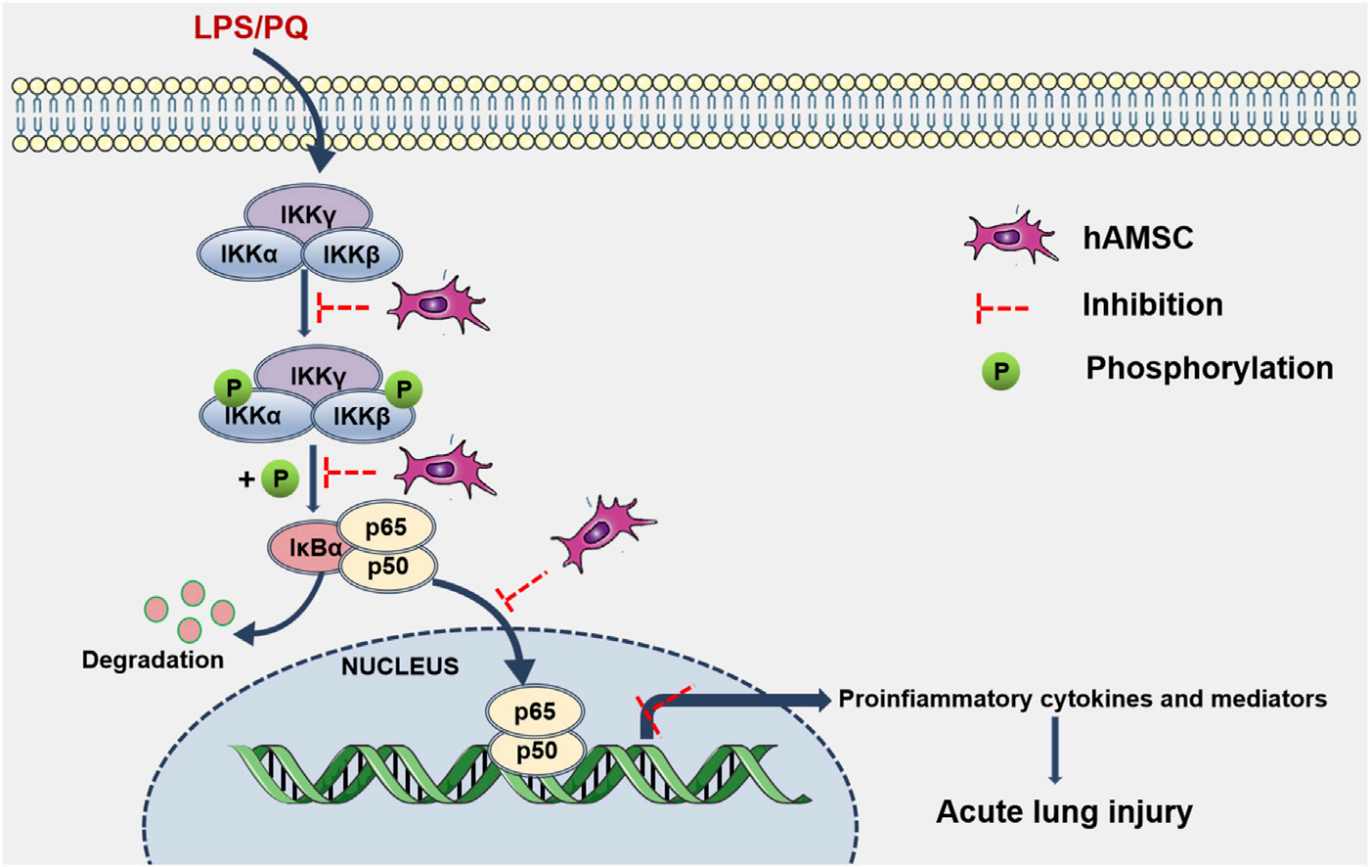
hAMSC inhibited the NF-κB signaling pathway. Schematic diagram shows that LPS/PQ can stimulate inflammatory responses and lung damage via the NK-κB signaling pathway. hAMSCs might inhibit the NK-κB signaling pathway, thereby blocking the inflammatory cascade and the progression of ALI.

In ALI/ARDS, neutrophil infiltration and inflammatory injury are landmark events (Abraham, 2003). The progression and severity of ALI (Butt et al., 2016) are closely related to the release of inflammatory factors and PMN infiltration in lung tissue. Our results showed that hAMSCs could inhibit the inflammatory response, reduce oxidative stress, and ameliorate lung injury. These inspiring results make hAMSCs a potential candidate for ALI treatment. Then, the mechanism of MSC therapy was discussed, which might involve multiple signaling pathways. Considering that LPS and PQ stimulate the production of proinflammatory factors and reactive oxygen nitrogen species (RONS), they may exacerbate inflammation and tissue damage via the NF-κB signaling pathway (Zhang et al., 2019a; Xiao et al., 2020; Zhang et al., 2020). In normal cells, p50 is inactive as a heterodimer with p65 (orc-Rel) through its interaction with inhibitory IκB proteins. When proinflammatory receptors activate the IKK complex, which consists of IKKα, IKKβ, and IKKγ, the IKK complex (mainly IKKβ) phosphorylates IκBα. Subsequently, ubiquitinated IκBα is degraded and activates the p50/p65 heterodimer. Once NF-κB is activated, it translocates to the nucleus and binds with promoter regions, inducing target transcription of inflammatory factors, cytokines, and chemokines (Wu et al., 2022). According to our results, p-IKKα/β, p-IκBα, and p-p65 levels were significantly attenuated in the hAMSC-transplanted groups, which was consistent with previous reports (Yang et al., 2011; Wang et al., 2016; Zhang et al., 2018; Zhang et al., 2020). Therefore, hAMSCs might inhibit the NK-κB pathway by blocking the inflammatory cascade and the progression of ALI (Figure 9).

## 5 Conclusion

Our results indicated that high-dose (HD) hAMSC treatment exerted beneficial therapeutic effects on two different ALI mouse models without detectable adverse reactions. The therapeutic effect of hAMSCs might involve NF-κB signaling pathway inhibition. Therefore, hAMSC treatment is a potential cell-based therapy for ALI.

## Data Availability

The raw data supporting the conclusions of this article will be made available by the authors, without undue reservation.

## Abbreviations

ALI, acute lung injury; ARDS, acute respiratory distress syndrome; BALF, bronchoalveolar lavage fluid; BCA, bicinchoninic acid; CFSE, carboxyfluorescein succinimidyl amino ester; ECMO, extracorporeal membrane oxygenation; FCM, flow cytometry; GMP, good manufacturing practice; hAMSCs, human amnion-derived mesenchymal stem cells; HSA, human serum albumin; H&E, hematoxylin and eosin; IF, immunofluorescence; MSCs, mesenchymal stem cells; LPS, lipopolysaccharide; PQ, Paraquat; PMN, polymorphonuclear; RONS, reactive oxygen nitrogen species; SOD, superoxide dismutase.

## References

[B1] Abe T. Madotto F. Pham T. Nagata I. Uchida M. Tamiya N. (2018). Epidemiology and patterns of tracheostomy practice in patients with acute respiratory distress syndrome in ICUs across 50 countries. Crit. Care 22 (1), 195. 10.1186/s13054-018-2126-6 30115127 PMC6097245

[B2] Abraham E. (2003). Neutrophils and acute lung injury. Crit. Care Med. 3, S195–S199. 10.1097/01.Ccm.0000057843.47705.E8 12682440

[B3] Bellani G. Laffey J. G. Pham T. Fan E. Brochard L. Esteban A. (2016). Epidemiology, patterns of Care, and mortality for patients with acute respiratory distress syndrome in intensive Care units in 50 countries. Jama 315 (8), 788–800. 10.1001/jama.2016.0291 26903337

[B4] Boyle A. J. O'Kane C. M. McAuley D. F. (2019). Where next for cell-based therapy in ARDS. Thorax 74 (1), 13–15. 10.1136/thoraxjnl-2018-212272 30420408

[B5] Butt Y. Kurdowska A. Allen T. C. (2016). Acute lung injury: A clinical and molecular review. Arch. Pathol. Lab. Med. 140 (4), 345–350. 10.5858/arpa.2015-0519-RA 27028393

[B6] Carbone A. Castellani S. Favia M. Diana A. Paracchini V. Di Gioia S. (2014). Correction of defective CFTR/ENaC function and tightness of cystic fibrosis airway epithelium by amniotic mesenchymal stromal (stem) cells. J. Cell Mol. Med. 18 (8), 1631–1643. 10.1111/jcmm.12303 24894806 PMC4190909

[B7] Cargnoni A. Romele P. Bonassi Signoroni P. Farigu S. Magatti M. Vertua E. (2020). Amniotic MSCs reduce pulmonary fibrosis by hampering lung B-cell recruitment, retention, and maturation. Stem Cells Transl. Med. 9 (9), 1023–1035. 10.1002/sctm.20-0068 32452646 PMC7445028

[B8] Cui P. Xin H. Yao Y. Xiao S. Zhu F. Gong Z. (2018). Human amnion-derived mesenchymal stem cells alleviate lung injury induced by white smoke inhalation in rats. Stem Cell Res. Ther. 9 (1), 101. 10.1186/s13287-018-0856-7 29650044 PMC5898065

[B9] Dinis-Oliveira R. J. Duarte J. A. Sánchez-Navarro A. Remião F. Bastos M. L. Carvalho F. (2008). Paraquat poisonings: Mechanisms of lung toxicity, clinical features, and treatment. Crit. Rev. Toxicol. 38 (1), 13–71. 10.1080/10408440701669959 18161502

[B10] Fan E. Brodie D. Slutsky A. S. (2018). Acute respiratory distress syndrome: Advances in diagnosis and treatment. Jama 319 (7), 698–710. 10.1001/jama.2017.21907 29466596

[B11] Gong L. Wang X. Xu S. Liao F. Zhou M. (2022). Human amnion-derived MSCs alleviate acute lung injury and hinder pulmonary fibrosis caused by paraquat in rats. Oxid. Med. Cell Longev. 2022, 3932070. 10.1155/2022/3932070 35345827 PMC8957415

[B12] Hass R. Kasper C. Böhm S. Jacobs R. (2011). Different populations and sources of human mesenchymal stem cells (MSC): A comparison of adult and neonatal tissue-derived MSC. Cell Commun. Signal 9, 12. 10.1186/1478-811x-9-12 21569606 PMC3117820

[B13] He F. Wang Y. Li Y. Yu L. (2020). Human amniotic mesenchymal stem cells alleviate paraquat-induced pulmonary fibrosis in rats by inhibiting the inflammatory response. Life Sci. 243, 117290. 10.1016/j.lfs.2020.117290 31923420

[B14] Johnson C. L. Soeder Y. Dahlke M. H. (2017). Concise review: Mesenchymal stromal cell-based approaches for the treatment of acute respiratory distress and sepsis syndromes. Stem Cells Transl. Med. 6 (4), 1141–1151. 10.1002/sctm.16-0415 28186706 PMC5442840

[B15] Liu H. Jiang C. La B. Cao M. Ning S. Zhou J. (2021a). Human amnion-derived mesenchymal stem cells improved the reproductive function of age-related diminished ovarian reserve in mice through Ampk/FoxO3a signaling pathway. Stem Cell Res. Ther. 12 (1), 317. 10.1186/s13287-021-02382-x 34078462 PMC8173966

[B16] Liu Q. W. Huang Q. M. Wu H. Y. Zuo G. S. Gu H. C. Deng K. Y. (2021b). Characteristics and therapeutic potential of human amnion-derived stem cells. Int. J. Mol. Sci. 22 (2), 970. 10.3390/ijms22020970 33478081 PMC7835733

[B17] Matute-Bello G. Downey G. Moore B. B. Groshong S. D. Matthay M. A. Slutsky A. S. (2011). An official American thoracic society workshop report: Features and measurements of experimental acute lung injury in animals. Am. J. Respir. Cell Mol. Biol. 44 (5), 725–738. 10.1165/rcmb.2009-0210ST 21531958 PMC7328339

[B18] Meyer N. J. Gattinoni L. Calfee C. S. (2021). Acute respiratory distress syndrome. Lancet 398 (10300), 622–637. 10.1016/s0140-6736(21)00439-6 34217425 PMC8248927

[B19] Monsel A. Hauw-Berlemont C. Mebarki M. Heming N. Mayaux J. Nguekap Tchoumba O. (2022). Treatment of COVID-19-associated ARDS with mesenchymal stromal cells: A multicenter randomized double-blind trial. Crit. Care 26 (1), 48. 10.1186/s13054-022-03930-4 35189925 PMC8860258

[B20] Papazian L. Aubron C. Brochard L. Chiche J. D. Combes A. Dreyfuss D. (2019). Formal guidelines: Management of acute respiratory distress syndrome. Ann. Intensive Care 9 (1), 69. 10.1186/s13613-019-0540-9 31197492 PMC6565761

[B21] Qin H. Zhao A. (2020). Mesenchymal stem cell therapy for acute respiratory distress syndrome: From basic to clinics. Protein Cell 11 (10), 707–722. 10.1007/s13238-020-00738-2 32519302 PMC7282699

[B22] Qin L. Zhang J. Xiao Y. Liu K. Cui Y. Xu F. (2022). A novel long-term intravenous combined with local treatment with human amnion-derived mesenchymal stem cells for a multidisciplinary rescued uremic calciphylaxis patient and the underlying mechanism. J. Mol. Cell Biol. 14 (2), mjac010. 10.1093/jmcb/mjac010 35142858 PMC9205756

[B23] Ranieri V. M. Rubenfeld G. D. Thompson B. T. Ferguson N. D. Caldwell E. Fan E. (2012). Acute respiratory distress syndrome: The berlin definition. Jama 307 (23), 2526–2533. 10.1001/jama.2012.5669 22797452

[B24] Ryan A. L. Ikonomou L. Atarod S. Bölükbas D. A. Collins J. Freishtat R. (2019). Stem cells, cell therapies, and bioengineering in lung biology and diseases 2017. An official American thoracic society workshop report. Am. J. Respir. Cell Mol. Biol. 61 (4), 429–439. 10.1165/rcmb.2019-0286ST 31573338 PMC6775946

[B25] Sun H. Jiang Y. Song Y. Zhang X. Wang J. Zhang J. (2020). The MUC5B mucin is involved in paraquat-induced lung inflammation. Oxid. Med. Cell Longev. 2020, 7028947. 10.1155/2020/7028947 32724493 PMC7381986

[B26] Tu C. Wang Z. Xiang E. Zhang Q. Zhang Y. Wu P. (2022). Human umbilical cord mesenchymal stem cells promote macrophage PD-L1 expression and attenuate acute lung injury in mice. Curr. Stem Cell Res. Ther. 17 (6), 564–575. 10.2174/1574888x17666220127110332 35086457

[B27] Wang J. Qin Y. Mi X. (2016). The protective effects of bone marrow-derived mesenchymal stem cell (BMSC) on LPS-induced acute lung injury via TLR3-mediated IFNs, MAPK and NF-κB signaling pathways. Biomed. Pharmacother. 79, 176–187. 10.1016/j.biopha.2016.02.037 27044826

[B28] Wilson J. G. Liu K. D. Zhuo H. Caballero L. McMillan M. Fang X. (2015). Mesenchymal stem (stromal) cells for treatment of ARDS: A phase 1 clinical trial. Lancet Respir. Med. 3 (1), 24–32. 10.1016/s2213-2600(14)70291-7 25529339 PMC4297579

[B29] Wu K. H. Li J. P. Chao W. R. Lee Y. J. Yang S. F. Cheng C. C. (2022). Immunomodulation via MyD88-nf?b signaling pathway from human umbilical cord-derived mesenchymal stem cells in acute lung injury. Int. J. Mol. Sci. 23 (10), 5295. 10.3390/ijms23105295 35628107 PMC9141460

[B30] Xiao K. He W. Guan W. Hou F. Yan P. Xu J. (2020). Mesenchymal stem cells reverse EMT process through blocking the activation of NF-κB and Hedgehog pathways in LPS-induced acute lung injury. Cell Death Dis. 11 (10), 863. 10.1038/s41419-020-03034-3 33060560 PMC7567061

[B31] Xuan Y. Y. Wu Y. Y. Xie Y. L. Chu J. G. Li G. X. Wang L. P. (2017). Human mesenchymal stem/stromal cells from human umbilical cord ameliorate acute respiratory distress syndrome in rats: Factors to consider. Crit. Care Med. 45 (7), e736–e737. 10.1097/ccm.0000000000002401 28622233

[B32] Yang H. Wen Y. Bin J. Hou-You Y. Yu-Tong W. (2011). Protection of bone marrow mesenchymal stem cells from acute lung injury induced by paraquat poisoning. Clin. Toxicol. (Phila) 49 (4), 298–302. 10.3109/15563650.2011.566882 21563905

[B33] Yang P. Humphrey S. J. Cinghu S. Pathania R. Oldfield A. J. Kumar D. (2019). Multi-omic profiling reveals dynamics of the phased progression of pluripotency. Cell Syst. 8 (5), 427–445. 10.1016/j.cels.2019.03.012 31078527 PMC6544180

[B34] Yip H. K. Fang W. F. Li Y. C. Lee F. Y. Lee C. H. Pei S. N. (2020). Human umbilical cord-derived mesenchymal stem cells for acute respiratory distress syndrome. Crit. Care Med. 48 (5), e391–e399. 10.1097/ccm.0000000000004285 32187077

[B35] Zhang F. Hu L. Wu Y. X. Fan L. Liu W. T. Wang J. (2019a). Doxycycline alleviates paraquat-induced acute lung injury by inhibiting neutrophil-derived matrix metalloproteinase 9. Int. Immunopharmacol. 72, 243–251. 10.1016/j.intimp.2019.04.015 31003001

[B36] Zhang L. C. Wang Y. Liu W. Zhang X. M. Fan M. Zhao M. (2018). Protective effects of SOD2 overexpression in human umbilical cord mesenchymal stem cells on lung injury induced by acute paraquat poisoning in rats. Life Sci. 214, 11–21. 10.1016/j.lfs.2018.10.020 30321544

[B37] Zhang L. Li Q. Liu W. Liu Z. Shen H. Zhao M. (2019b). Mesenchymal stem cells alleviate acute lung injury and inflammatory responses induced by paraquat poisoning. Med. Sci. Monit. 25, 2623–2632. 10.12659/msm.915804 30967525 PMC6474293

[B38] Zhang L. Wang Y. Shen H. Zhao M. (2020). Combined signaling of NF-kappaB and IL-17 contributes to Mesenchymal stem cells-mediated protection for Paraquat-induced acute lung injury. BMC Pulm. Med. 20 (1), 195. 10.1186/s12890-020-01232-5 32680482 PMC7367411

