# Supplementary material for: Human amnion-derived mesenchymal stem cells attenuate acute lung injury in two different acute lung injury mice models
Source: Front Pharmacol. 2023 Jun 14;14:1149659. doi: 10.3389/fphar.2023.1149659 (PMC10304826; doi:10.3389/fphar.2023.1149659)
Supplement: Supplementary file 1 [file Table1.docx]

Supplementary Material

Human amnion-derived mesenchymal stem cells attenuate acute lung injury in two different acute lung injury mice models

Yuxuan Wu^1†^, Hao Sun^1†^, Lianju Qin^2†^, Xiaomin Zhang^3^, Hao Zhou^1^, Yao Wang^1^, Lumin Wang^1^, Meng Li^1^, Jiayin Liu^2^^*^, Jinsong Zhang^1*^

^1^Department of Emergency, Jiangsu Province Hospital, The First Affiliated Hospital of Nanjing Medical University, Nanjing, Jiangsu 210029, China

^2^State Key Laboratory of Reproductive Medicine, Center of Clinical Reproductive Medicine, The First Affiliated Hospital of Nanjing Medical University, Nanjing, 210029, China

^3^Department of Emergency, Jiangnan University Medical Center, Wuxi, 214002, China

*** Correspondence:** Jiayin Liu: jyliu_nj@126.com; Jinsong Zhang: [zhangjso@njmu.edu.cn](mailto:zhangjso@njmu.edu.cn).

# Supplementary Data

## The preparation of hAMSCs

In this study, fifth-passage hAMSCs (hAMSCs/P5) were prepared and applied in all transplantation experiments. As previously reported, the expression of MSC-specific surface markers, the concentration of secreted cytokines in the cell culture supernatant, cell viability, bacterial contamination, and mycoplasma contamination of the cells were examined before hAMSC transplantation. Qualified hAMSCs were suspended in 1% human serum albumin (HSA) in 200 μL/tube containing a defined number of cells according to the different doses.

## ELISA

The levels of the cytokines IL-1β, IL-6, and TNF-α in the BALF were measured using mouse ELISA kits from R&D (MN, USA) according to the manufacturer's instructions.

## Quantitative real-time polymerase chain reaction

The lung homogenates were lysed with RNAiso plus (#9109, Takara Biotechnology), and total RNA was extracted. By using reverse transcription reagents (Takara Biotechnology, DRR037A), RNA was reverse-transcribed. Then, the expression of genes was measured by quantitative real-time PCR, which was performed on a StepOne real-time PCR system (Applied Biosystems, Foster City, CA) using SYBR Green Master Mix (Takara Biotechnology, DRR041A). All protocols were performed according to the manufacturer’s instructions. The primers used for quantitative real-time PCR analysis are listed in Table 1.

## Western blot analysis

To investigate the expression of proteins by Western blot analysis, lung tissue samples were lysed in RIPA lysis buffer containing protease and phosphatase inhibitors (Beyotime, Shanghai, China). The protein concentration of the samples was determined using a bicinchoninic acid (BCA) protein assay kit (Thermo Fisher Scientific, MA, United States). The samples were boiled for 10 min, and the proteins were separated by electrophoresis using a 10% sodium dodecyl sulfate (SDS)-polyacrylamide gel. After the proteins were transferred to a polyvinylidene difluoride membrane (PVDF, Millipore, Bedford, MA, United States), the membrane was incubated in blocking buffer [5% BSA in Tween-20 Tris-buffered saline (TBST)] for 2 h at ambient temperature and probed with various antibodies in blocking buffer overnight at 4°C. The membrane was washed four times with 0.1% TBST, probed with a secondary antibody in blocking buffer for 2 h at ambient temperature and then washed again with TBST. The membranes were detected with an enhanced chemiluminescence kit (Amersham Pharmacia Biotech, Piscataway, NJ, United States). The primary and secondary antibodies included those in the NF-κB Pathway Antibody Sampler Kit (#9936T, CST, Boston, USA) and β-actin (#8457, CST, Boston, USA).

# Supplementary Figures and Tables

## Supplementary Figures


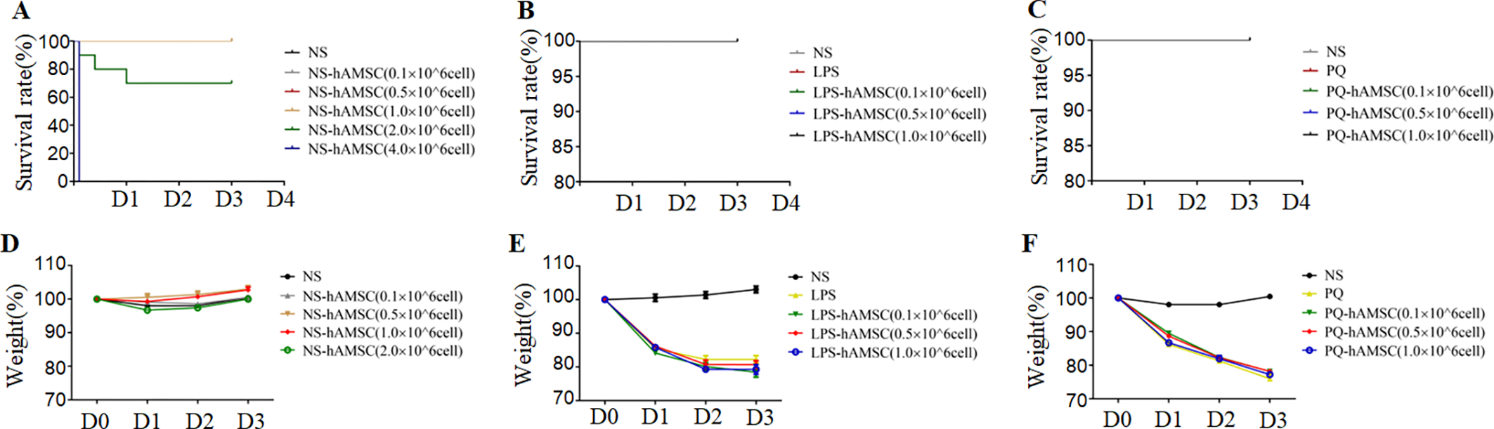


**Supplementary Figure 1.** **Safety assessment of hAMSCs.** (A-C) Survival rates of mice injected with hAMSCs in the different groups (A for normal mice, B for LPS-ALI mice model, C for PQ-LPS mice model). (D-F) Body weight changes of mice injected with hAMSCs in the different groups (D for normal mice, E for LPS-ALI mice model, F for PQ-ALI mice model).

## Supplementary Tables

| Species | Genes | sequence (5ʹ-3ʹ) |
| --- | --- | --- |
| mouse | β-actin | Forward: GGGAAATCGTGCGTGAC  Reverse: AGGCTGGAAAAGAGCCT |
| mouse | IL-1β | Forward: CAACCAACAAGTGATATTCTCCATG  Reverse: GATCCACACTCTCCAGCTGCA |
| mouse | IL-6 | Forward: TAGTCCTTCCTACCCCAATTTCC  Reverse: TTGGTCCTTAGCCACTCCTTC |
| mouse | TNF-ɑ | Forward: CACCACGCTCTTCTGTCT  Reverse: GGCTACAGGCTTGTCACTC |
